# Antioxidant, Anti-Inflammatory, and Chemical Composition Analysis of In Vitro *Huperzia serrata* Thallus and Wild *Huperzia serrata*

**DOI:** 10.3390/molecules31010195

**Published:** 2026-01-05

**Authors:** Yongchun Huang, Xinyuan Li, Liangfang Dai, Malong Cheng, Linlin Zhao, Yu Shen, Jiankun Xie, Xiangdong Luo

**Affiliations:** Jiangxi Provincial Key Laboratory of Biodiversity Conservation and Resource Utilization, College of Life Science, Jiangxi Normal University, Nanchang 330022, China; 18777076117@163.com (Y.H.); lxinyuann@163.com (X.L.); dailf79@163.com (L.D.); mlcheng86@163.com (M.C.); sheny202112@163.com (Y.S.);

**Keywords:** *Huperzia serrata*, in vitro thallus, HupA, antioxidant, metabolic analysis

## Abstract

Huperzine A is a preferred treatment option for Alzheimer’s disease. *Huperzia serrata* (Thunb. ex Murray) Trev. (*H. serrata*) has garnered significant attention for its ability to produce Huperzine A (HupA). However, natural populations of wild *H. serrata* (WH) are rapidly declining. Fortunately, our group obtained two types of *H. serrata* thalli (OT and ST) capable of stably producing Huperzine A, which have the potential to serve as an alternative resource to WH. To evaluate the feasibility of this strategy, we conducted a comprehensive assessment of both WH and *H. serrata* thallus. The results indicated that compared to WH, ST and OT exhibited stronger anti-inflammatory and antioxidant activities, with lower cytotoxicity. Notably, ST demonstrated a strong radical scavenging activity, reaching 93.23% (DPPH at 0.2 μg/mL) and 99.87% (ABTS at 4 μg/mL), and reduced nitrite production from 10.29 μM to 6.51 μM at 50 µg/mL. GC-MS and widely targeted metabolomics analyses revealed that the higher antioxidant and anti-inflammatory activities for ST and OT were due to higher concentrations of phenolic acids and flavonoids compared to WH. In addition, the HupA content in ST reached 36.56% of that found in WH. KEGG enrichment analysis revealed that the flavonoid, phenylalanine, and phenylpropanoid biosynthesis pathways may be involved in regulating the antioxidant activity. P-coumaroyl quinic acid and caffeoyl quinic acid are the crucial metabolites for antioxidant activity. These findings suggested that the *H. serrata* thallus could serve as a sustainable alternative to WH.

## 1. Introduction

Alzheimer’s disease (AD) is ranked as the third-leading cause of death. It is characterized as a common neurodegenerative disorder among the elderly [1]. The pathogenesis of AD is very complex, and effective treatment strategies are currently lacking [2]. Currently, the “cholinergic hypothesis” suggests that the most effective approach to alleviating AD symptoms is to inhibit the activity of acetylcholinesterase [3,4]. Numerous research studies have confirmed that HupA is one of highly efficient, strongly specific, and reversible acetylcholinesterase inhibitors, characterized by low toxicity and unique pharmacological properties [5]. These advantages suggest that HupA possesses significant potential for AD treatment. In America, HupA has been approved as a nutritional supplement for alleviating AD symptoms [4,6].

In 1986, researchers successfully obtained HupA from *Huperzia serrata* (Thunb. ex Murray) Trev. (*H. serrata*), a traditional Chinese herb [7]. Wild *H. serrata* (WH) is used in its whole-plant form in traditional Chinese medicine for the treatment of various conditions, such as pain relief, hemostasis, detoxification, dehumidification, and heat clearing [8]. However, WH grows very slowly and exhibits low reproductive capacity under natural conditions. It takes 6 to 15 years for *H. serrata* to develop from a gametophyte into a mature individual, rendering large-scale cultivation impractical [5]. Recently, many scientists have attempted to address the shortage of HupA resources via chemical synthesis and endophyte fermentation. However, chemically synthesized HupA demonstrates low activity, high toxicity, and a high cost [9]. Many endophytic fungi isolated from the host plant of WH would lose their ability to produce HupA after several subcultures [10,11]. As a result, the endangered WH and its related species remain the primary source of HupA, making WH increasingly rare and expensive for HupA production. Therefore, exploring innovative approaches for the sustainable utilization of *H. serrata* is of paramount importance.

Plant tissue culture is a highly effective method for the conservation and in vitro propagation of rare plant germplasms. Although in vitro micropropagation studies of *H. serrata* were initiated as early as 1957, progress has been significantly hindered by its abundant endophytes and difficulties in regeneration [12]. Fortunately, our group successfully obtained several genotypic *H. serrata* thalli capable of stably producing HupA [5,13]. *H. serrata* thalli exhibited advantages such as a high survival rate and a short propagation cycle, requiring only 85 days of subculturing to reach peak HupA accumulation [14]. The original *H. serrata* thallus was treated with H_2_O_2_, yielding a mutant with a more than twofold increase in HupA production [15]. After more than ten subcultures, the HupA yield remained consistently stable. Therefore, compared to chemical synthesis and endophyte fermentation, the *H. serrata* thallus offers a more stable and convenient route for obtaining HupA.

Recently, researchers have explored various strategies to enhance the HupA content in the *H. serrata* thallus. The impact of exogenous additives on HupA accumulation was evaluated [16], and the relationship between thallus morphology and HupA content was investigated [13]. Genes associated with the HupA biosynthesis pathway were identified via combined analyses of transcriptomic and widely targeted metabolomics [17], and several key genes, including *HsPKS1*, were subsequently cloned [18]. These studies provided promising strategies for alleviating the resource shortage of *H. serrata* [13].

However, efforts at evaluating the potential of *H. thallus* as a substitute for WH cannot focus solely on its HupA production capacity. Other bioactivities are critically important, particularly the antioxidant and anti-inflammatory properties, which play a crucial role in mitigating the oxidative stress and inflammatory responses associated with neurodegenerative diseases [19]. Furthermore, cytotoxicity is a key safety parameter. To enable a comprehensive comparison, a thorough analysis of the chemical profiles in both WH and the cultivated thallus is essential. Therefore, we employed an in vitro bioactivity assay, GC-MS, and widely target metabolomics analysis to evaluate the original *H. serrata* thallus (OT), high-yield HupA mutant (ST), and WH. These detailed results will provide valuable insights and establish a crucial foundation for further investigation and practical applications of the *H. serrata* thallus.

## 2. Results

### 2.1. HupA Content in Three H. serrata Materials

**Figure 1:**
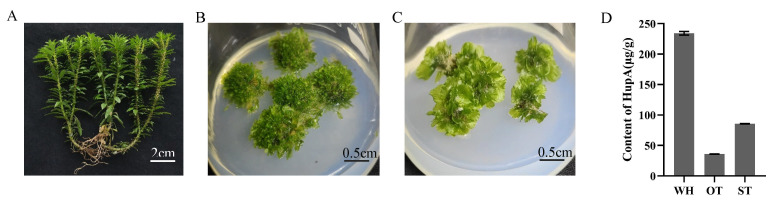
The morphological character of three materials. (**A**) wild *H. serrata* (WH). (**B**) The original *H. serrata* thallus (OT). (**C**) The mutant of *H. serrata* thallus (ST). (**D**) HupA content in the three materials.

WH exhibits dichotomous branching from its apical meristem, with each branch bearing gemmae and displaying clear differentiation into roots, stems, and leaves (Figure 1A). In contrast, the *H. serrata* thallus lacks such organ differentiation (Figure 1B,C). Furthermore, the HupA content varied significantly among the three *H. serrata* samples (Figure 1D), with WH showing the highest level at 234.21 ± 2.97 μg/g, followed by ST at 85.63 ± 0.35 μg/g, and OT at 35.89 ± 0.17 μg/g.

### 2.2. Antioxidant Capacity in H. serrata Thallus and Wild Type

#### 2.2.1. 2,2-Diphenyl-1-picrylhydrazyl (DPPH) Radical Scavenging Activity

**Figure 2:**
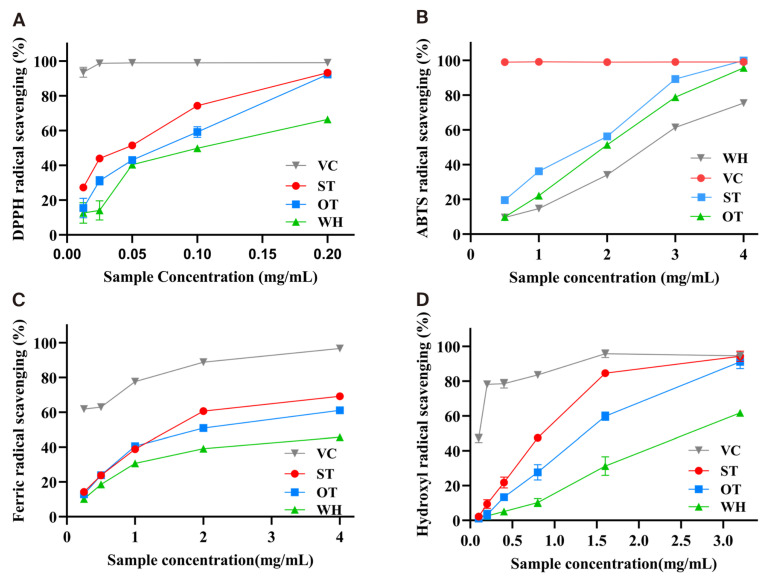
Antioxidant activity assays of the three materials’ crude extracts. (**A**) The DPPH radical scavenging rate. (**B**) The ABTS radical scavenging rate. (**C**) The Ferric reducing antioxidant power. (**D**) The Hydroxyl radical (OH^−^) scavenging rate.

The DPPH assay is a common method to detect the antioxidant activity, and the DPPH radical scavenging rate can reflect the antioxidant capacity. Ascorbic acid (VC) was used as the positive control. As shown in Figure 2A, ST, OT, and WH showed a concentration-dependent effect on DPPH radical scavenging. When the crude extract concentration reached 0.2 mg/mL, DPPH radical scavenging activity reached 93.23% (ST), 92.30% (OT), and 66.44% (WH). As listed in Table 1, the IC_50_ values were determined to be 0.05 ± 0.01 mg/mL, 0.07 ± 0.01 mg/mL, and 0.10 ± 0.01 mg/mL, correspondingly. The results revealed that ST and OT possessed more significant antioxidant potential than WH.

#### 2.2.2. ABTS Radical Scavenging Activity Assay

The ABTS scavenging ability of three samples exhibited a positive association with the concentration ranging from 0.5 to 4 mg/mL (Figure 2B). When the concentration reached to 4 mg/mL, the ABTS radical scavenging activities of ST and OT were 99.87% and 95.66%, respectively, higher than that of WH (75.43%). In addition, the IC_50_ values of ST and OT were 1.69 ± 0.01 mg/mL and 1.95 ± 0.02 mg/mL, while the IC_50_ value of WH was 2.59 ± 0.02 mg/mL (Table 1). The results suggested that ST and OT exhibited a more superior ABTS scavenging ability compared to WH.

#### 2.2.3. Ferric (Fe^3+^) Reducing Antioxidant Power

Assessment of the Fe^3+^ reducing power was conducted via the IC_50_ value, with a lower value indicating stronger reducing power. As showed in Figure 2C, all samples exhibited a dose-dependent relationship. The IC_50_ values for crude extracts of ST, OT, and WH were 1.51 ± 0.01, 1.91 ± 0.02, and 5.00 ± 0.42 mg/mL, respectively, indicating that ST and OT possess superior reducing activity compared to WH (Table 1).

#### 2.2.4. Hydroxyl Radical (OH^−^) Scavenging Rate Analysis

The results indicated that, at the same concentration, ST and OT are significantly superior WH in OH^−^ scavenging. When the concentration was to 3.2 mg/mL, the OH^−^ scavenging capacity of WH reached 61.76%, while those of ST and OT were 94.31% and 91.08%, respectively (Figure 2D). Meanwhile, the IC_50_ values were 0.85 ± 0.02 mg/mL, 1.35 ± 0.07 mg/mL, and 2.57 ± 0.14 mg/mL (Table 1).

### 2.3. Total Phenolic Content in H. serrata Thallus and Wild Type

The total phenolic content was assessed using a calibration curve (y = 0.08892 + 1.70126x, R_2_ = 0.99465) for gallic acid (0 to 0.64 mg/mL) and expressed in gallic acid equivalents (GAE) per gram dry extract weight. In methanol extracts of three samples, the total phenolic content ranged from 20.99 to 60.09 mg GAE/g, showing a nearly threefold variation. Among the three extracts of *H. serrata*, ST had the highest phenolic content (60.09 ± 1.04 mg GAE/g), while OT and WH had a lower phenolic content (27.65 ± 1.28 and 20.99 ± 1.64 mg GAE/g, respectively) (Table 2). The total phenolic content of the three extracts of *H. serrata* corresponded to the results of metabolome analysis. Compared to WH, the *H. serrata* thallus showed a higher total phenolic content, which probably can be attributed to its higher content of flavonoids and phenolic acids.

### 2.4. Cytotoxicity in H. serrata Thallus and Wild Type

**Figure 3:**
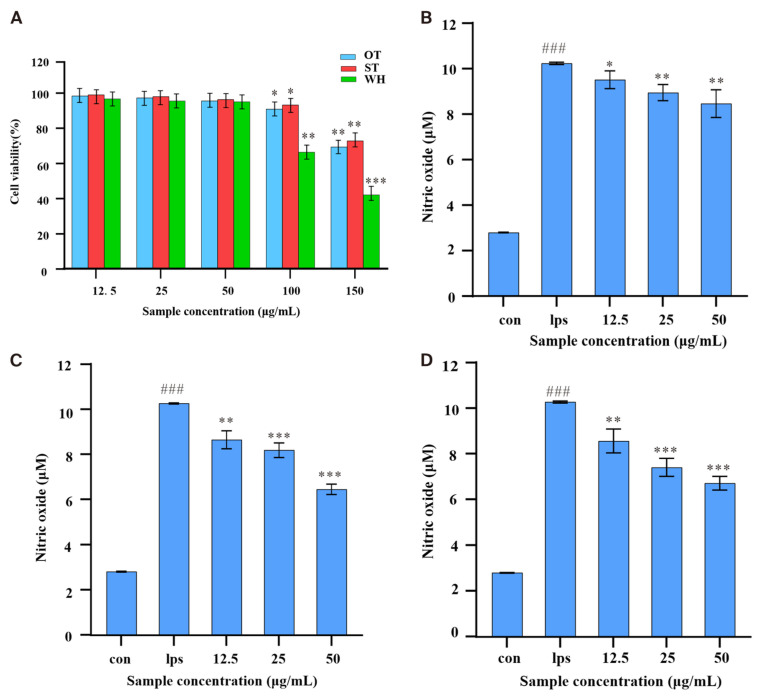
Cytotoxicity and anti-inflammatory activity assays of three *H. serrata* crude extracts. (**A**) RAW 264.7 cell viability. (**B**) The effect of OT on NO production in LPS-treated RAW 264.7 cells. (**C**) The effect of ST on NO production. (**D**) The effect of WH on NO production. Data was shown as the mean ± SD of the three replicates. Statistical significance compared to the blank control group is indicated as follows: * *p* < 0.05, ** *p* < 0.01, *** *p* < 0.001; significance of the LPS group compared to the blank control is specifically denoted as ^###^ *p* < 0.001.

In this study, the MTT method was employed for detecting the impact of methanol extract of three materials on the cell viability of RAW 264.7 cells. As shown in Figure 3A, when the concentration of crude extract was ≤50 μg/mL, the cell survival rates of the three samples were higher than 95%. When the concentration reached 100 μg/mL, the cell survival rates of the ST, OT, and WH groups were 93.29%, 90.88%. and 66.45%, respectively. At the 200 μg/mL concentration, the cell survival rates were less than 72.87%. The results indicated that the three samples had no clear inhibition effect on cell viability at low concentrations (≤50 μg/mL). However, when the concentration increased, the cell survival rate for the WH group was the lowest, and ST and OT had significantly less cytotoxicity.

### 2.5. Anti-Inflammatory Capability in H. serrata Thallus and Wild Type

To test the anti-inflammatory effect of the methanol extract of *H. serrata*, the Griess method was used to determine the release of NO in LPS-induced RAW 264.7 cells. Since lower levels of *H. serrata* extracts (≤50 µg/mL) had no clear inhibitory influence on the viability of RAW 264.7 cells, *H. serrata* extracts at concentrations of 12.5, 25, and 50 µg/mL were subsequently used to test their anti-inflammatory effects in vitro. As shown in Figure 3B–D, the NO concentration was 2.81 µM for the control group, while in the LPS-induced group, it was 10.29 µM, which was nearly a 4-fold increase compared to the control. In the treatment groups, however, all three samples gradually reduced LPS-stimulated NO production in a concentration-dependent pattern, and different samples had different degrees of inhibition on NO release from LPS-stimulated RAW 264.7 cells. At 50 µg/mL, OT, ST, and WH inhibited NO by 14.38% (8.81 µM), 36.73% (6.51 µM), and 33.82% (6.81 µM), respectively. This result indicated that ST, OT, and WH had significant anti-inflammatory activity, and the ST extract demonstrating the highest activity (NO production reducing from 10.29 μM to 6.51 μM, with an inhibition rate of 36.73% at 50 µg/mL).

### 2.6. Compositional Analysis of Three H. serrata Types by GC-MS

Eighty-four compounds were detected in the three samples by GC-MS analysis (shown in Supplementary Table S1, Supplementary Figure S1). These compounds could be classified into 12 classes, including flavonoids, terpenoids, ketones, esters, etc. (Table 3). Of the compounds, 42 and 40 were detected in ST and OT, respectively, and 41 compounds in WH. Moreover, there were notable differences in the content of some compounds among the three types of *H. serrata.* For example, alkaloids and phenols were higher in WH. Flavonoids, terpenoids, and esters were higher in OT and ST than in WH (Table 3). These compounds exhibited strong associations with antioxidant activity.

Further analysis indicated that the kinds and contents of potentially toxic compounds were different in the three types of *H. serrata* on the basis of a database query (https://organchem.csdb.cn/scdb/default.asp, accessed on 16 May 2025). It showed that 17 toxic compounds (39.79%) were detected in WH, while there were 11 toxic compounds in OT (22.50%) and 13 toxic compounds in ST (32.51%) (Table 4). This verified that OT and ST had lower toxicity. Digitoxose (13.2%) and 2-Methoxy-4-vinylphenol (9.34%), which are lipids and phenols, respectively, are the most abundant toxic compounds in WH. 4H-Pyran-4-one, 2,3-dihydro-3,5-dihydroxy-6-methyl (8.17%) and pentanoic acid (7.04%), which are ketones and carboxylic acids, are the most abundant toxic compounds in ST. The highest contents of toxic compounds detected in OT were for pentanoic acid (6.2%) and benzofuran, 7-cyclohexyl-2,3-dihydro-2-methyl (4.6%), which are carboxylic acids and aldehydes.

### 2.7. Compositional Analysis of Three H. serrata Types by UPLC-MS/MS

Our research employed UPLC-MS/MS-based widely targeted metabolomics to analyze metabolites in three *H. serrata*. To assess the overall differences between sample groups, we performed Orthogonal Partial Least Squares–Discriminant Analysis (OPLS-DA). The OPLS-DA revealed high similarity in metabolic profiles among samples within the same group, while significant differences were observed between different groups (Supplementary Figure S2A). A permutation test with 200 iterations yielded a Q^2^ value of 0.999, demonstrating the high reliability of the established model (Supplementary Figure S2B).

**Figure 4:**
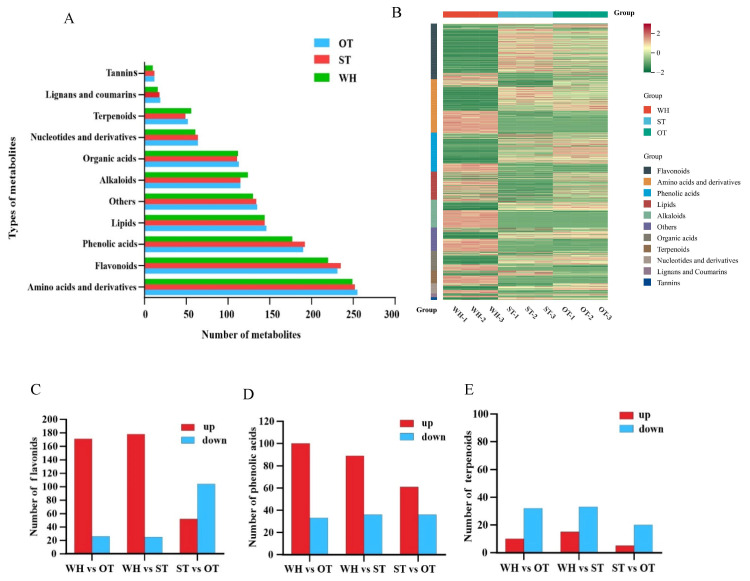
Widely targeted metabolome analysis of three *H. serrata.* (**A**) The classification results of different metabolites in the three groups. (**B**) Cluster heatmap of metabolite abundance. (**C**) The number difference in flavonoids among different groups. (**D**) The number difference in phenolic acids among different groups. (**E**) The number difference in terpenoids among different groups.

Finally, a total of 1376 compounds were characterized through metabolomic analysis, which can be categorized into 11 different classes (Figure 4A). Compound analysis revealed that phenolic acids, derivatives, flavonoids, and amino acids were the primary compounds in *H. serrata*, with each type containing over 175 compounds in number. Then, lipids, others, alkaloids, and organic acids followed, with each of them containing approximately 110–150 compounds. The number of tannins, lignans, and coumarins was the lowest, with fewer than 65 compounds of each type. Further analysis showed that the types and quantities of compounds are very similar among the WH, ST, and OT. The number of compounds of each type only differs by 2~6 compounds among the three samples. Compared with WH, there are more types of flavonoids and phenolic acids in ST and OT, with fewer alkaloids and terpenoids. These results are consistent with our GC-MS analysis. Notably, HupA was detected in three *H. serrata* materials, with the highest content found in the wild-type plants.

For WH vs. ST, WH vs. OT, and ST vs. OT comparisons, the former was the control group and the latter the experimental group, with up-/down-regulation indicating metabolites in the experimental group compared to the control.

According to the relative concentrations of metabolites, we conducted a hierarchical heatmap cluster analysis on the three types of *H. serrata* (Figure 4B). Analysis of the clustering heatmap of metabolite abundance showed that there were notable differences in metabolite content among the three samples. The contents of organic acids, terpenoids, amino acids, and their derivatives, lipids and alkaloids, were higher in WH. Meanwhile, the content of flavonoids, tannins, and phenolic acids was higher in ST and OT compared to WH. Many studies have demonstrated that flavonoids and phenolic acids exhibit various biological effects, such as antioxidant and anti-inflammatory effects [20], which again demonstrates the higher chemical activity and antioxidant capacity of OT and ST. These results were also consistent with the GC-MS results.

The results of the widely targeted metabolome analysis showed that the *H. serrata* thallus contains many active ingredients, including flavonoids, phenolic acids, terpenoids, etc. Variable Importance in the Projection (VIP) > 1.0 and a fold change value (FC) ≥ 2 or FC ≤ 0.5 were used as criteria to screen the differential metabolites of flavonoids, phenolic acids, and terpenoids. In the WH vs. ST group, there were 203 differential metabolites in flavonoids, 125 differential metabolites in phenolic acids, and 48 differential metabolites in terpenoids. In the WH vs. OT group, there were 197 differential metabolites in flavonoids, 133 differential metabolites in phenolic acids, and 42 differential metabolites in terpenoids (shown in Supplementary Table S2). Compared with WH, there were 87.68% flavonoids, 71.20% phenolic acids, and 31.25% terpenoids whose expression was up-regulated in ST. Compared with WH, there were 86.80% flavonoids, 75.19% phenolic acids, and 23.81% terpenoids up-regulated in OT (Figure 4C–E). Among the flavonoids, 23 quercetin derivatives and 19 cases of kaempferol and its derivatives were found in the *H. serrata* thallus. Compared with WH, five new quercetin derivatives, namely quercetin 3-O-(6″-acetyl-glucoside), quercetin-3-O-robinobioside, quercetin-3-O-(6″-O-acetyl) galactoside 7,3′,4′-Trihydroxyquercetin and quercetin-3-O-rhamnoside (Quercitrin), and a new kaempferol derivative, kaempferol-6,8-di-C-glucoside-7-O-glucoside, were discovered in the *H. serrata* thallus. The results suggested that compared with WH, the number of phenolic acids and flavonoids in ST and OT was higher, further confirming that ST and OT demonstrated a superior antioxidant activity and anti-inflammatory activity compared to WH.

### 2.8. KEGG Enrichment Analysis of Antioxidant-Related Differential Metabolism

**Figure 5:**
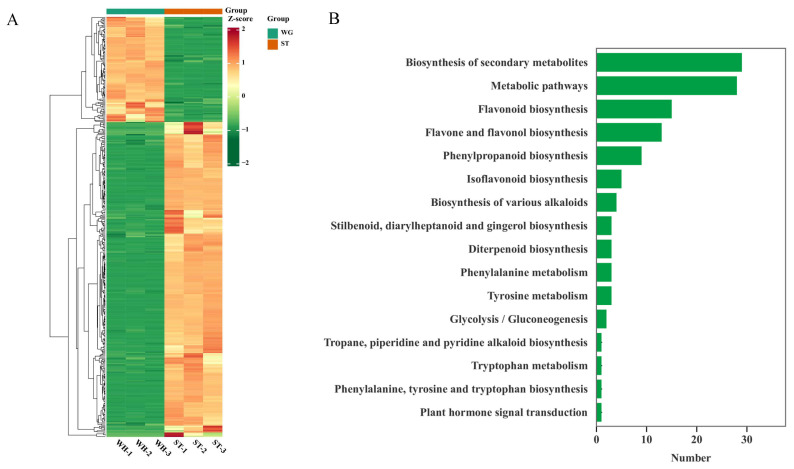
KEGG enrichment analysis of three *H. serrata.* (**A**) Statistical histogram of KEGG pathway number of flavonoids, phenolic acids, and terpenoids. (**B**) Cluster heatmap of flavonoids, phenolic acids, and terpenoids’ metabolites.

According to the biological activity experiments on the three materials, ST had the strongest antioxidant capacity and WH had the lowest. Therefore, ST and WH were selected for a correlation analysis of antioxidant biosynthesis. Since flavonoids, phenolic acids, and terpenoids have relatively good antioxidant activity, we focused on these three types of compounds. The heatmap indicated that most of these compounds were up-regulated in ST (Figure 5A). Kyoto Encyclopedia of Genes and Genomes (KEGG) enrichment analysis [21] was conducted on the detected flavonoids, phenolic acids, and terpenoids to screen out antioxidant-related metabolites. As shown in Figure 5B, a total of 377 compounds were enriched in 16 pathways. The results revealed that the two pathways with the highest number of enriched metabolites were biosynthesis of secondary metabolites (ko01110) and metabolic pathways (ko01100). However, these two pathways are fundamental metabolic pathways and are not representative of antioxidants. Instead, the pathways associated with antioxidant were the flavonoid biosynthesis pathway (ko00941), the phenylalanine metabolism pathway (ko00360), and the phenylpropanoid biosynthesis pathway (ko00940). These results suggested that the antioxidant mechanism of *H. serrata* is highly likely to be mediated by flavonoid, phenylalanine and phenylpropanoid biosynthesis pathway.

### 2.9. Analysis of Antioxidant-Related Metabolic Pathways

**Figure 6:**
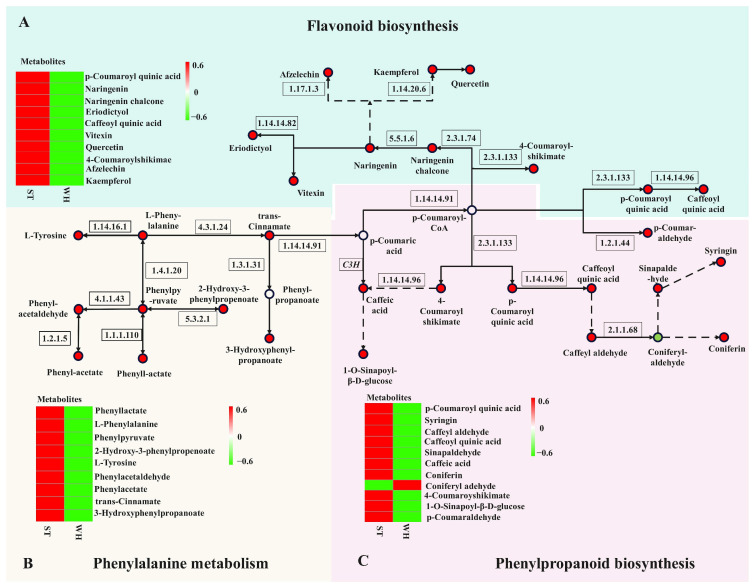
Antioxidant metabolic pathway map in *H. serrata*. (**A**) Flavonoid biosynthesis pathway, (**B**) phenylalanine metabolic pathway, and (**C**) phenylpropanoid biosynthesis pathway. Circles stand for the metabolites (green shows down-regulation; red shows up-regulation).

According to KEGG enrichment pathway analysis, a total of three metabolic pathways related to antioxidants were enriched, which were flavonoid biosynthesis (ko00941), phenylalanine metabolism (ko00360), and phenylpropanoid biosynthesis (ko00940). By integrating the metabolic pathway map of antioxidants and analyzing metabolomics data, 30 metabolites were detected, which were participating in the biosynthesis pathways of flavonoids, phenolic acids, and terpenoids. Among these metabolites, 29 were up-regulated in ST, while 1 was down-regulated (Figure 6). These findings indicate that compared with WH, the content of flavonoids, phenolic acids, and terpenoids in ST was much higher. Consequently, the anti-inflammatory and antioxidant effects of ST were higher than those of WH. This finding corresponded to the results obtained by antioxidant activity assays.

Twenty-one metabolites were identified in phenylpropanoid and flavonoid biosynthetic pathways. Among these, caffeoyl quinic acid and P-coumaroyl quinic acid were the most different metabolites in these two pathways, with log_2_FC values of 10.96 and 11.95. All nine differentially accumulated metabolites detected by the phenylalanine metabolism pathway were up-regulated in ST, with phenylacetate showing the highest log_2_FC value of 8.74, making it the most different metabolite in this pathway. It is suggested that these three metabolites are likely to function as the key differential metabolites in the antioxidant metabolic pathway of *H. serrata.*

## 3. Discussion

*H. serrata* is a very important traditional Chinese herb in the Lycopodiaceae family, possessing significant economic value [22,23]. However, wild *H. serrata* is endangered in China owing to its slow growth rates, overharvesting, and habitat destruction [24]. Some other species, such as *Phlegmariurus tetrastichus* (syn. *Huperzia tetrasticha*) [25] and *Huperzia squarrosa* (G. Forst.) Trevis [26], are also capable of producing HupA. However, they are more difficult to obtain and are rarer in nature than *H. serrata*, making them less ideal as natural sources of HupA [12]. Therefore, given the rapid decline in wild *H. serrata* resources*,* the *H. serrata* thallus is crucial for ensuring the drug supply and sustainable development of *H. serrata*. In vitro micropropagation of *H. serrata* offers several advantages, including controllable cultivation conditions, a shorter growth period, and a rapid reproductive rate [5]. In this study, the chemical compositions and medicinal value of *H. serrata* thallus were thoroughly evaluated. Previous studies have demonstrated that alkaloids, triterpenes, and flavonoids are the primary bioactive constituents in wild *H. serrata* [27], and our study further confirmed this conclusion. These active substances are known for their diverse pharmacological properties, including neuroprotective, anti-inflammatory, and anti-tumor effects. Our results showed that there was no significant difference in the types of compounds among the different *H. serrata* with variations in their content. These results validate the potential of the thallus as a viable substitute for wild *H. serrata*.

Medicinal plants with antioxidant properties can be used to treat pathological processes such as aging, behavioral and psychiatric disorders, cancer, atherosclerosis, and rheumatoid arthritis, which involve free radicals [28]. Antioxidant experiments in this study revealed that the antioxidant activity of ST and OT exceeded that of WH. This superiority is likely attributed to the higher concentrations of flavonoids and phenolic acids in the *H. serrata* thallus compared to WH, as these compounds are known for their antioxidant properties. Our results of widely targeted metabolomics analysis showed that more than 80% of flavonoids and 70% of phenolic acids in ST and OT were up-regulated metabolites compared to WH. Moreover, among the 30 metabolites detected in the antioxidant pathway, 29 metabolites were up-regulated in ST. These findings suggest that flavonoids and phenolic acids in the *H. serrata* thallus are more likely to be synthesized and accumulated, which also endows the *H. serrata* thallus with higher antioxidant activity. Furthermore, compared with WH, the *H. serrata* thallus exhibit lower toxicity, suggesting its potential as an effective and safe alternative medicinal resource.

Flavonoids are natural antioxidants. They not only directly alleviate oxidative stress through metal ion chelating and ROS scavenging but also exert indirect effects by activating antioxidant enzymes and suppressing pro-oxidant enzymes [29]. Due to their outstanding antioxidant activity, flavonoids are utilized in the cosmetics, nourishment, and pharmaceutical industries [30]. Among them, quercetin and kaempferol are two major diet-derived flavonoids, accounting for 70% of flavonoid intake in the human diet and playing a pivotal role in human health [31]. Regarding quercetin derivatives, 23 quercetin derivatives have been identified in the *H. serrata* thallus, including quercetin-3-O-glucoside (isoquercetin), quercetin-3,3′-dimethyl ether, and so on. Compared with WH, five new quercetin derivatives were found in the *H. serrata* thallus, including quercetin-3-O-robinobioside, 7,3′,4′-Trihydroxyquercetin, quercetin 3-O-(6″-acetyl-glucoside), quercetin-3-O-(6″-O-acetyl) galactoside, and quercetin-3-O-rhamnoside (quercitrin). Similarly, there were 19 kaempferol compounds, and their derivatives were detected in the *H. serrata* thallus. Compared with WH, kaempferol-6,8-di-C-glucoside-7-O-glucoside, a new kaempferol derivative, was found in the *H. serrata* thallus. These compounds demonstrate strong antioxidant activity, highlighting the antioxidant potential of the *H. serrata* thallus. Our results also indicated that the flavonoids in *H. serrata* are mainly produced through the flavonoid biosynthesis pathway, which also involves the phenypropanoid metabolic pathway and can lead to a range of phenolic acids as secondary metabolite [32]. Analysis of the log_2_FC value revealed that caffeoyl quinic acid and P-coumaroyl quinic acid were the most significantly differentiated metabolites in the two pathways. This result indicated that caffeoyl quinic acid and P-coumaroyl quinic acid may be the key metabolites in the antioxidant pathway of *H. serrata*.

The inflammatory response facilitates the clearance of invading immunogens. However, excessive inflammation may lead to chronic diseases and even mortality [33]. LPS is a component of Gram-negative bacterial outer membranes [34]. LPS could activate macrophages via TLR 4 (toll-like receptor 4) to release pro-inflammatory mediators and inflammation-associated enzymes, thereby enhancing bactericidal activity [35,36]. Therefore, we established LPS-induced RAW 264.7 cells as a cell inflammatory model to assess the inhibitory activity of inflammation of the three *H. serrata*. Each experimental group had significant inhibitory effects, with ST exhibiting a superior performance compared to the other two groups. Furthermore, our results indicated that the three *H. serrata* had a higher dose-dependent anti-inflammatory activity. Moreover, our study detected many anti-inflammatory compounds. For example, 3-acetylursolic acid compounds have anticancer, anti-inflammatory, anti-ulcer, antibacterial, and antiviral activities [37,38]. Hup A exhibits good anti-inflammatory activity [39]. Furthermor, flavonoids such as calycosin-7-O-glucosides and acacetin-7-O-galactoside also have anti-inflammatory activities [40,41]. Furthermore, the syringin, salicylic acid, and phenolic acid compounds also exhibit anti-inflammatory activity [42,43]. The higher contents of these compounds in the *H. serrata* thallus suggested that the *H. serrata* thallus may possess enhanced anti-inflammatory properties.

## 4. Materials and Methods

### 4.1. Plant Materials

The wild plant of *H. serrata* (WH) used in this study was collected from Nanping, Fujian, China. The newly harvested spore-bearing stems of WH were inoculated onto 6,7V medium containing 0.5 mg/L IAA for thallus induction, yielding OT, which exhibited lower HupA accumulation. Subsequent subculturing was performed on SH medium containing 0.5 mg/L NAA. ST was obtained as a mutant derived from OT following exposure to 3% H_2_O_2_ for 16 min, which induced DNA-level mutations [15]. The samples were authenticated by Professor ZR Zou, a botanist of Jiangxi Normal University. Both ST and OT exhibit a stable production of HupA, but the HupA content in ST is higher [5,15]. ST and OT are cultured under 13 h of light and 11 h of darkness at 24 ± 1 °C, with a light intensity of 2000 Lux.

### 4.2. Preparation of H. serrata Extract

The stems and leaves of WH, together with the whole *H. serrata* thallus, were utilized as raw materials for the preparation of extracts. Clean samples were taken from the medium, washed, and then dried in an oven at 40 °C for 2 d. The method of sample preparation was modified according to previous methods [44]. About 10 g powdered *H. serrata* was extracted in a round-bottomed flask containing 150 mL of analytical-grade methanol. After extracting twice by the condensation reflux method, the mixture was filtered through filter paper and then concentrated with a rotary evaporator. The methanol extract of each sample with three replicates was freeze-dried to powdered form and stored at −20 °C.

### 4.3. HupA Extraction and Content Analysis

HupA extraction and quantification were performed based on established methods with modifications [15]. The cleaned materials were dried in an oven at 40 °C to a constant weight, then ground into a uniform powder using a mortar and pestle for subsequent use. Then, the powdered samples (1 g) were treated with 20 mL of 2% tartaric acid, followed by incubation (55 °C, 22 h) with periodic agitation, ultrasonication (100 MHz, 45 °C, 30 min), and centrifugation (5500 rpm, 25 °C, 15 min). The supernatant was collected, and the pellet was re-extracted twice with 14 mL of tartaric acid. Combined supernatants were adjusted to pH 9–10 with ammonia and concentrated to dryness at 40 °C. Subsequently, the supernatants was sequentially extracted under sealed conditions: first, with 4 mL of methanol under sealed conditions for 20 h, and then with 3 mL of methanol under sealed conditions for 2 h, followed by three cycles of extraction with 2 mL of methanol for 1 h each. The collected extracts were diluted to 5 mL with methanol, and they were filtered through a 0.22 μm membrane prior to HupA quantification.

An Agilent high-performance liquid chromatography system (1260 Infinity II) was employed, equipped with a Waters XTerra^®^ MSC18 column (purchased from Waters Company, Milford, MA, USA) with dimensions of 4.6 mm × 250 mm and a 5.0 μm particle size. The mobile phase consisted of solvent A: chromatographic-grade methanol, and solvent B: ammonium acetate solution (0.08 mol·L^−1^, pH 6.00), with a ratio of A:B = 3:7. The flow rate was set at 0.8 mL/min, the elution time was 60 min, the detector used was ultraviolet light (308 nm), and the column temperature was maintained at 25 °C.

### 4.4. Antioxidant Activity Assay

#### 4.4.1. DPPH Radical Scavenging Activity

The DPPH assay is a common method used to detect the antioxidant activity, and the DPPH radical scavenging rate can reflect the antioxidant capacity. The DPPH radical scavenging ability of methanol extracts of the three samples was determined via the standard method [45]. In sum, the extracts with concentrations of 0.0125, 0.025, 0.05, 0.1, and 0.2 mg/mL were mixed with 1.5 mL of DPPH solution, followed by a 30 min incubation under dark conditions. Then, the absorbance of the reaction mixture was measured at 517 nm. In a blank group, DPPH was replaced with 1.5 mL of methanol. The reagent blank group consisted of 1.5 mL methanol and 1.5 mL DPPH.

#### 4.4.2. ABTS Radical Scavenging Activity

When exposed to potassium persulfate, a strong oxidizing agent, ABTS forms a stable green free radical. ABTS radical scavenging activity analysis was performed according to the method of previous studies [46]. Firstly, 7 mM ABTS (aqueous solution) was mixed with 2.45 mM potassium persulfate at a 1:1 ratio and maintained under dark conditions for 12 to 16 h at room temperature. The ABTS radical working solution was prepared by adding 1 mL of ABTS to 19 mL of ethanol, with the absorbance adjusted to about 0.70 ± 0.05 at 734 nm. Then, about 1.9 mL ABTS working solution was mixed with 0.2 mL samples and kept under dark conditions for 6 min at 37 °C, followed by measuring the absorbance at 734 nm.

#### 4.4.3. OH^−^ Scavenging Activity

The assessment of OH^−^ scavenging activity was measured according to the previous method [47] with minor modifications. Briefly, the extracts with concentrations of 0.1, 0.2, 0.4, 0.8, 1.6, and 3.2 mg/mL were mixed with 1 mL of FeSO_4_ (0.75 mM), 1 mL of phenanthroline alcohol solution (0.75 mM), 2 mL of phosphate-buffered solution (0.2 mM PBS), 1 mL of H_2_O_2_ solution (0.03%), and 1 mL of deionized water. After incubation at 37 °C for 1 h, the absorbance was detected at 512 nm.

#### 4.4.4. Fe^3+^ Reducing Antioxidant Power Assay

Fe^3+^ reducing antioxidant power is closely associated with antioxidant activity [46]. Compounds possessing reducing properties usually act as electron donors that are capable of reducing the oxidized intermediates generated during lipid peroxidation processes, thereby functioning as primary or secondary antioxidants [48]. The assessment method for the ability to reduce Fe^3+^ was according to Mohamed et al. [49]. Here, 1 mL of sample solution was mixed with 1 mL of distilled water and 1 mL of potassium ferricyanide (1%, *w*/*v*). After 20 min incubation at 50 °C, 1 mL of ferric chloride solution (0.1%, *w*/*v*) and 1 mL of trichloroacetic acid (10%, *w*/*v*) were added. Finally, the absorbance was tested at 700 nm.

The percentages of scavenging effects were calculated based on the subsequent equations [50,51]: ABTS and DPPH radical scavenging rate (%) = [1 − (Ai − Aj)/Ao] × 100%. OH^−^ scavenging rate (%) and the ability to reduce Fe^3+^ (%) = (Ai − Ao)/(Aj − Ao) × 100%.

Here, Ao represents the absorbance of the control group, Ai is the absorbance of the samples, and Aj represents the absorbance of the reagent blank. To eliminate interference from the sample’s inherent absorbance, we introduced Aj. Each concentration was tested in triplicate, and a positive control (ascorbic acid) was treated under the same conditions as the samples.

### 4.5. Total Phenolic Content Assay

The assessment method for determining the total phenolic content of *H. serrata* extract was performed according to Zong et al. [52]. We accurately pipetted 0.1 mL of standard sample, added 0.5 mL of Folin phenol (0.1 mol) reagent, reacted in dark for 6 min, added 1.4 mL of 7.5% Na_2_CO_3_ solution, mixed well, and reacted for 30 min at room temperature in darkness. We accurately pipetted 0.1 mL of the sample to be tested. The method was the same as that of the standard with deionized water standard solution as the blank control groups. Then, the absorbance values were recorded at 760 nm. We performed each experiment in triplicate.

### 4.6. Cytotoxicity and Anti-Inflammatory Activity Analysis

RAW264.7 cells possess active phagocytic capabilities and can effectively internalize exogenous substances such as nanoparticles, making them an ideal model for studying immune cell-mediated toxic responses. Cell viability was assessed using the classic MTT assay, a method that indirectly reflects the viable cell number via mitochondrial succinate dehydrogenase activity. The MTT assay is noted for its simple procedure, low cost, and applicability to high-throughput screening. The murine macrophage cell line RAW 264.7 was derived from the tumor induced by the Abelson murine leukemia virus (purchased from Suzhou Haixing Biotechnology Co., Ltd., Suzhou, Jiangsu, China). RAW 264.7 cells were cultured in DMEM containing 10% fetal bovine serum (FBS) and 100 U/mL penicillin–streptomycin at 37 °C in a 5% CO_2_ atmosphere. The cells in the logarithmic growth stage were blown into a single cell suspension with a dropper. DMEM containing 10% FBS was added until the cell concentration rose to 1 × 10^5^ pieces/mL. We seeded 100 μL/well into 96-well plates, with triplicate wells for each group, and we cultured overnight. Next, we removed the old medium, and the other groups were given the corresponding concentration of drug-containing medium except the blank group, cultured for 24 h. We removed the supernatant and added 10 μL MTT (5 mg·mL^−1^) and 90 μL DMEM. After 4 h, the supernatant was gently aspirated using a syringe, and 150 μL of DMSO was added, followed by a 30 min incubation. The absorbance was measured with a microplate reader at 540 nm and repeated three times.

The cell concentration of a RAW 264.7 single-cell suspension was adjusted to 5 × 10^5^ cells/mL by adding it into DMEM containing 10% FBS. Subsequently, the cells were seeded into 24-well plates and cultured overnight. The supernatant was randomly divided into a lipopolysaccharide (LPS) group, sample group, and blank group. All groups except the blank control group were pretreated with LPS (2 μg/mL). After 2 h, the sample group was added with different concentrations of drugs. We took 100 μL supernatant, added 50 μL Griess reagent solution A and 50 μL Griess reagent solution B, avoided light color, and incubated for 10 min. The absorbance values were detected at 450 nm with a microplate reader.

### 4.7. Analysis of Components by GC-MS

The chemical components of *H. serrata* extract were evaluated by Thermo’s GC-MS analysis system Trace1300/ISQ (purchased from Thermo Scientific Company, Waltham, MA, USA). The extract was mixed with 1 mL methanol and filtered through a 0.45 μm Whatman nylon syringe filter. Three biological replicates were prepared for each group. The sample (1 μL aliquots) was injected into the GC-MS at 250 °C in a shunt mode (10:1) with a carrier gas (>99.999% helium, the flow rate of 1 mL/min), separated by the HP-5 MS capillary column (30 m × 0.25 mm, 0.25 µm). The temperature program commenced at 40 °C, followed by a linear ramp at 4 °C/min until reaching 210 °C, and then it was maintained at 210 °C for 5 min. Subsequently, the temperature was increased to 250 °C at 4 °C/min and held for 15 min. Finally, the temperature was raised to 280 °C at the same heating rate and stabilized for 1 min. Mass spectrometry conditions: electron impact ionization source (EI); ion source temperature, 230 °C; quadrupole temperature, 150 °C; MS interface temperature, 280 °C; electron energy, 70 eV; scan mode, selected ion monitoring (SIM); qualitative and quantitative ions were accurately scanned in accordance with GB 23200.8-2016 [53].

Compound identification was performed by comparison with the National Institute of Standards and Technology (NIST) database, with only those exhibiting ≥80% similarity to the reference compounds in NIST selected.

### 4.8. Widely Targeted Metabolic Profiling

Ultra-performance liquid chromatography tandem mass spectrometry (UPLC-MS/MS) enables accurate identification and quantification. Based on the widely targeted metabolic analysis, the three materials were vacuum freeze-dried, crushed into a powder, weighed to 50 mg, and then dissolved in 1.2 mL 70% methanol extract. The samples underwent 30 s vortex mixing at 30 min intervals, and this process was repeated 6 times. We centrifuged (12,000 rpm, 3 min), took the supernatant, filtered the supernatant with a 0.22 um filter membrane, and stored it in a sampling bottle for subsequent UPLC-MS/MS analysis.

The analysis was conducted at Mai Wei Metabolism Company (Wuhan, Hubei, China). The sample extracts were analyzed using an UPLC-ESI-MS/MS system. Chromatographic separation was performed using an ExionLC™ AD UPLC system (purchased from SCIEX Company, Framingham, MA, USA). Mass spectrometric detection was carried out with an Applied Biosystems 6500 Q TRAP mass spectrometer (purchased from SCIEX Company, Waltham, MA, USA). The analytical conditions were as follows. UPLC: column, Agilent SB-C18 (purchased from Agilent Technologies, Santa Clara, CA, USA, 1.8 µm, 2.1 mm × 100 mm); the mobile phase consisted of solvent A, pure water with 0.1% formic acid, and solvent B, acetonitrile with 0.1% formic acid. Sample measurements were performed with a gradient program that employed the starting conditions of 95% A, 5% B. Within 9 min, a linear gradient to 5% A, 95% B was programmed, and a composition of 5% A, 95% B was kept for 1 min. Subsequently, a composition of 95% A, 5.0% B was adjusted within 1.1 min and kept for 2.9 min. The flow velocity was set as 0.35 mL per minute; the column oven was set to 40 °C; the injection volume was 2 μL. The effluent was alternatively connected to an ESI-triple quadrupole-linear ion trap (QTRAP)-MS. The ESI source operation parameters were as follows: source temperature 500 °C; ion spray voltage (IS) 5500 V (positive ion mode)/−4500 V (negative ion mode); ion source gas I (GSI), gas II(GSII), and curtain gas (CUR) were set at 50, 60, and 25 psi, respectively; and the collision-activated dissociation (CAD) was high. QQQ scans were acquired as MRM experiments with collision gas (nitrogen) set to medium. The DP (declustering potential) and CE (collision energy) for individual MRM transitions were determined, with further DP and CE optimization. A specific set of MRM transitions was monitored for each period according to the metabolites eluted within this period.

### 4.9. Widely Targeted Metabolomic Data Analysis

For two-group analysis, differential metabolites were determined by VIP (VIP > 1) and absolute Log2FC (|Log2FC| ≥ 1.0). VIP values were extracted from OPLS-DA results, which also contain score plots and permutation plots, as generated using R package MetaboAnalystR (version 4.0). The data was log transformed (log2) and mean centered before OPLS-DA. In order to avoid overfitting, a permutation test (200 permutations) was performed.

Identified metabolites were annotated using the KEGG Compound database (http://www.kegg.jp/kegg/compound/, accessed on 25 April 2025), and annotated metabolites were then mapped to the KEGG Pathway database (http://www.kegg.jp/kegg/pathway.html, accessed on 25 April 2025). Pathways mapped to with significantly regulated metabolites were then fed into MSEA (metabolite sets enrichment analysis), and their significance was determined by the hypergeometric test’s *p*-values.

### 4.10. Data Statistical Analysis

All the experiments were performed in triplicate. SPSS 20 statistical software was employed to conduct one-way analysis of variance (ANOVA) for the differences among the groups.

## 5. Conclusions

In summary, compared to WH, the *H. serrata* thallus exhibited significantly higher anti-inflammatory and antioxidant activity, along with lower cytotoxicity. In addition, there was no remarkable difference in the types of compounds among the three *H. serrata*, but there was a significant difference in content. Further investigation revealed that flavonoid, phenylalanine, and phenylpropanoid biosynthesis pathways may be involved in regulating the antioxidant activity for *H. serrata*. Among these, caffeoyl quinic acid and P-coumaroyl quinic acid were recognized as the crucial metabolites. These results indicated that the *H. serrata* thallus could be an alternative medicinal resource for WH. Furthermore, the data from this research provide useful information for the further investigation and utilization of the *H. serrata* thallus.

## Figures and Tables

**Table 1:** IC_50_ values of antioxidant capabilities in three *H. serrata*.

Assay Method	Sample Types (mg/mL)			
	VC	OT	ST	WH
DPPH	-	0.07 ± 0.01 ^b^	0.05 ± 0.01 ^c^	0.10 ± 0.01 ^a^
ABTS^+^	-	1.95 ± 0.02 ^b^	1.69 ± 0.01 ^c^	2.59 ± 0.02 ^a^
FRAP	0.10 ± 0.01	1.91 ± 0.02 ^b^	1.51 ± 0.01 ^c^	5.00 ± 0.42 ^a^
OH^−^	0.11 ± 0.01	1.35 ± 0.07 ^b^	0.85 ± 0.02 ^c^	2.57 ± 0.14 ^a^

Values are expressed as mean ± SEM (*n* = 3). Mean values within a row with different superscript letters are significantly different (*p* < 0.05) as determined by one-way ANOVA. The scavenging rates of DPPH and ABTS for VC were nearly 100%, so the IC_50_ values could not be calculated.

**Table 2:** Total phenolic contents in the three plant materials expressed in terms of gallic acid equivalent (mg GAE/g).

Plant Materials	Phenolic Contents (mg GAE/g)
ST	60.09 ± 1.04 ^a^
OT	27.65 ± 1.28 ^b^
WH	20.99 ± 1.64 ^c^

Means (±standard error) with different superscripts in each column are significantly different at *p* < 0.05.

**Table 3:** Classification statistics of identified compounds by GC-MS.

Compound Type	OT		ST		WH	
	Kind	Area %	Kind	Area %	Kind	Area %
Flavonoid	4	6.62	5	9.49	2	4.27
Terpenoid	4	9.00	3	7.96	1	2.28
Ketone	5	8.20	6	18.46	7	11.88
Ester	7	26.60	8	16.80	7	14.09
Hydrocarbon	5	6.69	5	6.37	1	2.59
Carboxylic Acid	4	12.79	5	11.02	8	19.31
Alkaloid	2	1.92	2	2.51	4	3.92
Aldehyde	2	6.15	3	4.73	2	14.57
Alcohol	4	5.98	3	4.72	4	3.36
Ether	1	4.60	1	6.39	1	2.97
Phenol	1	0.49	1	0.98	3	12.11
Saccharide	1	0.58	—	—	2	13.93

**Table 4:** Toxic compounds identified in the three *H. serrata* by GC-MS.

RT/min	Compounds	Relative Content (%)		
		OT	ST	WH
10.13	Pentanoic acid	6.20	7.04	-
11.50	M-Cymene	0.52	1.1	-
12.16	Benzeneacetaldehyde	-	0.51	-
12.38	Acetone	2.01	2.96	-
13.40	Prolintane	2.12	-	-
13.42	Thymine	-	3.1	2.98
13.90	Phenol, 2-methoxy-	-	-	1.46
15.69	4H-Pyran-4-one, 2,3-dihydro-3,5-dihydroxy-6-methyl	3.55	8.17	0.92
17.17	5-Hydroxy-2-(hydroxymethyl)-4H-pyran-4-one	-	0.99	-
18.85	5-Hydroxymethylfurfural	2.49	3.57	1.37
20.94	Thymol	-	0.98	-
21.63	2-Methoxy-4-vinylphenol	-	-	9.34
25.14	4-Methylimidazolidine-2-thione	-	-	1.29
26.33	Digitoxose	-	-	13.2
34.87	Myristic acid	-	-	0.42
37.44	Pentadecanoic acid	-	-	0.71
38.00	Phytol, acetate	0.88	0.69	0.44
38.94	Pyrimidine, 2,4-diamino-6-ethyl-5-phenyl-	-	1.92	-
39.44	Palmitoleic acid	-	-	1.23
39.52	Benzenepropanoic acid, 3,5-bis(1,1-dimethylethyl)-4-hydroxy-, methyl ester	0.8	0.83	0.43
40.96	cis-5,8,11,14,17-Eicosapentaenoic acid	0.57	-	-
42.71	Lycopodine	-	-	1.3
43.57	Phytol	2.87	-	2.28
46.14	Allylestrenol	-	-	0.52
46.63	Lycodoline	-	-	1.48
51.56	Phorbol-12,13-dihexanoate	-	0.65	-
60.82	Lupulon	-	-	0.42
78.75	Vitamin E	0.49	-	-

## Data Availability

The original contributions presented in this study are included in the article/Supplementary Material. Further inquiries can be directed to the corresponding author.

## Supplementary Materials

The following supporting information can be downloaded at https://www.mdpi.com/article/10.3390/molecules31010195/s1, Figure S1: GC-MS analysis; Figure S2: (A) OPLS-DA analysis of three *H. serrata*. (B) A permutation test of OPLS-DA analysis; Table S1: GC-MS Analysis Result; Table S2: LC-MS/MS Analysis Result.

## References

[1] Twarowski B., Herbet M. (2023). Inflammatory processes in Alzheimer’s disease-pathomechanism, diagnosis and treatment: A review. Int. J. Mol. Sci..

[2] Li J., Wang L., Zhang X., Shi J., Zhu Y., Wang H., Zhu X., Zhu Q., Luo J.L. (2025). Translating Alzheimer’s disease mechanisms into therapeutic opportunities. Biomolecules.

[3] Howes M.R., Fang R., Houghton P.J. (2017). Effect of Chinese herbal medicine on Alzheimer’s disease. Int. Rev. Neurobiol..

[4] Xiao Y., Liang W., Liu D., Zhang Z., Chang J., Zhu D. (2022). Isolation and acetylcholinesterase inhibitory activity of asterric acid derivatives produced by *Talaromyces aurantiacus* FL15, an endophytic fungus from *Huperzia serrata*. 3 Biotech.

[5] Yang Y., Dai L., Wu D., Dong L., Tu Y., Xie J., Luo X. (2021). In vitro propagation, Huperzine A content and antioxidant activity of three genotypic *Huperzia serrata*. Plants.

[6] Damar U., Gersner R., Johnstone J.T., Schachter S., Rotenberg A. (2016). Huperzine A as a neuroprotective and antiepileptic drug: A review of preclinical research. Expert Rev. Neurother..

[7] Barbosa Filho J.M., Medeiros K.C.P., Diniz M.d.F.F.M., Batista L.M., Athayde-Filho P.F., Silva M.S., da Cunha E.V.L., Almeida J.R.G.S., Quintans-Júnior L.J. (2006). Natural products inhibitors of the enzyme acetylcholinesterase. Rev. Bras. Farmacogn..

[8] Chu Z., Sun Q., Mao M., Wu Y., Yu L., Xu J., Liu K., Qin L., Zhu B. (2025). Pharmacology, phytochemistry, and traditional uses of *Huperzia serrata* (Thunb. ex Murray) Trev. Fitoterapia.

[9] Ma X., Tan C., Zhu D., Gang D.R., Xiao P. (2007). Huperzine A from *Huperzia* species—An ethnopharmacolgical review. J. Ethnopharmacol..

[10] Cruz-Miranda O.L., Folch-Mallol J., Martínez-Morales F., Gesto-Borroto R., Villarreal M.L., Taketa A.C. (2020). Identification of a Huperzine A-producing endophytic fungus from *Phlegmariurus taxifolius*. Mol. Biol. Rep..

[11] Jiang F., Qi B., Ding N., Yang H., Jia F., Luo Y., Wang J., Liu X., Wang X., Tu P. (2019). Lycopodium alkaloids from *Huperzia serrata*. Fitoterapia.

[12] Ma X., Gang D.R. (2008). In vitro production of huperzine A, a promising drug candidate for Alzheimer’s disease. Phytochemistry.

[13] Ji Z., Tu Y., Ding M., Chen X., Jiang X. (2014). In vitro culture of *Huperzia serrata* thallus for the medicinal component production. Nat. Prod. Res. Dev..

[14] Ji Z., Tu Y., Chen M., Ye L. (2016). Conditional optimization and kinetic research on producing huperzine A using *Huperzia serrata* in vitro. Chin. Tradit. Herbal Drugs.

[15] Ye L., Tu Y., Huang Q., Yu X., Yuan H. (2017). Analysis on the progeny diversity of H_2_O_2_ induced mutation on *Huperzia serrata* culture in vitro. Chin. Med. Mat..

[16] Ye Y., Tu Y., Yu X., Huang Q., Yuan H. (2022). Different response of culturing thallus of *Huperzia serrata* in vitro to exogenous lysine and aspartic acid. Genom. Appl. Biol..

[17] Wu H., Shen Y., Zou F., Yao S., Chen Y., Yang H., Luo X. (2024). Combined transcriptome and widely targeted metabolome analysis reveals the potential mechanism of HupA biosynthesis and antioxidant activity in *Huperzia serrata*. Front. Plant. Sci..

[18] Shen Y., Wu H., Wu D., Yao S., Li X., Dai L., Luo X. (2024). Cloning and expression analysis of *HsPKS1* gene related to synthesis of Huperzine A in *Huperzia serrata*. Chin. Tradit. Herbal Drugs.

[19] Xie J., Su R., Wu D., Qin Y., Yun X. (2021). A novel synthetic compound shows antioxidant and anti-inflammatory activity and alleviates cognitive deficits in rats for the treatment of Alzheimer’s disease. Ann. Palliat. Med..

[20] Kuljarusnont S., Iwakami S., Iwashina T., Tungmunnithum D. (2024). Flavonoids and other phenolic compounds for physiological roles plant species delimitation, and medical benefits: A promising view. Molecules.

[21] Kanehisa M., Sato Y., Kawashima M., Furumichi M., Tanabe M. (2016). KEGG as a reference resource for gene and protein annotation. Nucleic Acids Res..

[22] Pan W.J., Miao L.Y., Fan S.P., Lv P.W., Lin A.H., Geng H., Song F.J., Zhang P. (2023). New insights into the composition and diversity of endophytic bacteria in cultivated *Huperzia serrata*. Can. J. Microbiol..

[23] Jiang S., Gao B.B., Ou Y.F., Zhao Q.S. (2024). Lycopodium alkaloids from *Huperzia serrata* and their cholinesterase inhibitory activities. Phytochemistry.

[24] Shen Z., Liu X., Yang J., Wang Y., Yao K., Huo Q., Fu Y., Wei Y., Guo B. (2022). The temporal and spatial endophytic fungal community of *Huperzia serrata*: Diversity and relevance to huperzine A production by the host. BMC Microbiol..

[25] Nett R.S., Dho Y., Low Y.Y., Sattely E.S. (2021). A metabolic regulon reveals early and late acting enzymes in neuroactive Lycopodium alkaloid biosynthesis. Proc. Natl. Acad. Sci. USA.

[26] Cuthbertson D., Piljac-Žegarac J., Lange B.M. (2012). Validation of a microscale extraction and high-throughput UHPLC-QTOF-MS analysis method for huperzine A in *Huperzia*. Biomed. Chromatogr..

[27] Cao D., Sun P., Bhowmick S., Wei Y., Guo B., Wei Y., Mur L.A.J., Sun Z. (2021). Secondary metabolites of endophytic fungi isolated from *Huperzia serrata*. Fitoterapia.

[28] Ralte L., Khiangte L., Thangjam N.M., Kumar A., Singh Y.T. (2022). GC-MS and molecular docking analyses of phytochemicals from the underutilized plant, *Parkia timoriana* revealed candidate anti-cancerous and anti-inflammatory agents. Sci. Rep..

[29] Naksuriya O., Daowtak K., Tima S., Okonogi S., Mueller M., Toegel S., Khonkarn R. (2022). Hydrolyzed flavonoids from *Cyrtosperma johnstonii* with superior antioxidant, antiproliferative, and anti-inflammatory potential for cancer prevention. Molecules.

[30] Dias M.C., Pinto D.C.G.A., Silva A.M.S. (2021). Plant flavonoids: Chemical characteristics and biological activity. Molecules.

[31] Su P., Li Z., Yan X., Wang B., Bai M., Li Y., Xu E. (2024). Quercetin and Kaempferol inhibit HMC-1 activation via SOCE/NFATc2 signaling and suppress hippocampal mast cell activation in lipopolysaccharide-induced depressive mice. Inflamm. Res..

[32] Nabavi S.M., Šamec D., Tomczyk M., Milella L., Russo D., Habtemariam S., Suntar I., Rastrelli L., Daglia M., Xiao J. (2020). Flavonoid biosynthetic pathways in plants: Versatile targets for metabolic engineering. Biotechnol. Adv..

[33] Kwon M., Lee J., Park S., Kwon O.H., Seo J., Roh S. (2020). Exopolysaccharide isolated from *Lactobacillus plantarum* L-14 has anti-inflammatory effects via the toll-like receptor 4 pathway in LPS-induced RAW 264.7 cells. Int. J. Mol. Sci..

[34] Liu J., Kang R., Tang D. (2024). Lipopolysaccharide delivery systems in innate immunity. Trends Immunol..

[35] Bai R., Guo J. (2025). SPI1 upregulated LILRB2 to enhance the immunosuppressive phenotype of LPS-tolerant macrophages by inhibiting TLR8-mediated MyD88/NF-κB signaling. Biol. Direct.

[36] Kim Y.S., Kim E.K., Nawarathna W.P.A.S., Dong X., Shin W.B., Park J.S., Moon S.H., Park P.J. (2017). Immune-stimulatory effects of *Althaea rosea* flower extracts through the MAPK signaling pathway in RAW264.7 cells. Molecules.

[37] Dzubak P., Hajduch M., Vydra D., Hustova A., Kvasnica M., Biedermann D., Markova L., Urban M., Sarek J. (2006). Pharmacological activities of natural triterpenoids and their therapeutic implications. Nat. Prod. Rep..

[38] Jain N.K., Chandrasekaran B., Khazaleh N., Jain H.K., Lal M., Joshi G., Jha V. (2025). Computational network pharmacology, molecular docking, and molecular dynamics to decipher natural compounds of *Alchornea laxiflora* for liver cancer chemotherapy. Pharmaceuticals.

[39] Chen X., Zhang Y., Cao Z., Wang Y., Liao M., Guan Y., Zhu C., Wang W., Huang W., Li W. (2024). Huperzine A targets Apolipoprotein E: A potential therapeutic drug for diabetic nephropathy based on omics analysis. Pharmacol. Res..

[40] Chen S., Liu J., Dong G., Zhang X., Liu Y., Sun W., Liu A. (2021). Flavonoids and caffeoylquinic acids in *Chrysanthemum morifolium* Ramat flowers: A potentially rich source of bioactive compounds. Food Chem..

[41] Yu D.H., Bao Y.M., An L.J., Yang M. (2009). Protection of PC12 cells against superoxide-induced damage by isoflavonoids from *Astragalus mongholicus*. Biomed. Environ. Sci..

[42] Dong H., Wu M., Wang Y., Du W., He Y., Shi Z. (2021). Total syntheses and anti-inflammatory activities of syringin and its natural analogues. J. Nat. Prod..

[43] Rosheen, Sharma S., Utreja D. (2023). Salicylic Acid: Synthetic strategies and their biological activities. ChemistrySelect.

[44] Bonamigo T., Campos J.F., Oliveira A.S., Torquato H.F.V., Balestieri J.B.P., Cardoso C.A.L., Paredes-Gamero E.J., de Picoli Souza K., Dos Santos E.L. (2017). Antioxidant and cytotoxic activity of propolis of *Plebeia droryana* and *Apis mellifera* (Hymenoptera, Apidae) from the Brazilian Cerrado biome. PLoS ONE.

[45] Elufioye T.O., Chinaka C.G., Oyedeji A.O. (2019). Antioxidant and anticholinesterase activities of *Macrosphyra Longistyla* (DC) Hiern relevant in the management of Alzheimer’s disease. Antioxidants.

[46] Ismail N.Z., Arsad H., Samian M.R., Hamdan M.R. (2017). Determination of phenolic and flavonoid contents, antioxidant activities and GC-MS analysis of *Clinacanthus nutans* (Acanthaceae) in different locations. Agrivita J. Agric. Sci..

[47] Gulcin İ. (2020). Antioxidants and antioxidant methods: An updated overview. Arch. Toxicol..

[48] Brewer M.S. (2011). Natural antioxidants: Sources, compounds, mechanisms of action, and potential applications. Compr. Rev. Food Sci. Food Saf..

[49] Mohamed W.A.S., Ismail N.Z., Omar E.A., Abdul Samad N., Adam S.K., Mohamad S. (2020). GC-MS evaluation, antioxidant content, and cytotoxic activity of propolis extract from peninsular malaysian stingless bees, *Tetrigona apicalis*. Evid.-Based Complement. Altern. Med..

[50] Wang Z., Jia S., Cui J., Qu J., Yue Y., Sun Q., Zhang H. (2019). Antioxidant activity of a polysaccharide produced by *Chaetomium globosum* CGMCC 6882. Int. J. Biol. Macromol..

[51] Yang S., Li X., Zhang H. (2024). Ultrasound-assisted extraction and antioxidant activity of polysaccharides from *Tenebrio molitor*. Sci. Rep..

[52] Zong L., Zhang J., Dai L., Liu J., Yang Y., Xie J., Luo X. (2020). The Anti-inflammatory properties of *Rhododendron molle* leaf extract in LPS-induced RAW264.7. Chem. Biodivers..

[53] (2016). Food Safety National Standard—Determination of 500 Pesticides and Related Chemical Residues in Fruits and Vegetables—Gas Chromatography-Mass Spectrometry.

