# Supplementary material for: Antioxidant, Anti-Inflammatory, and Chemical Composition Analysis of In Vitro Huperzia serrata Thallus and Wild Huperzia serrata
Source: Molecules. 2026 Jan 5;31(1):195. doi: 10.3390/molecules31010195 (PMC12787719; doi:10.3390/molecules31010195)
Supplement: Supplementary file 1 [file molecules-31-00195-s001.zip › Supplementary Figures.pdf]

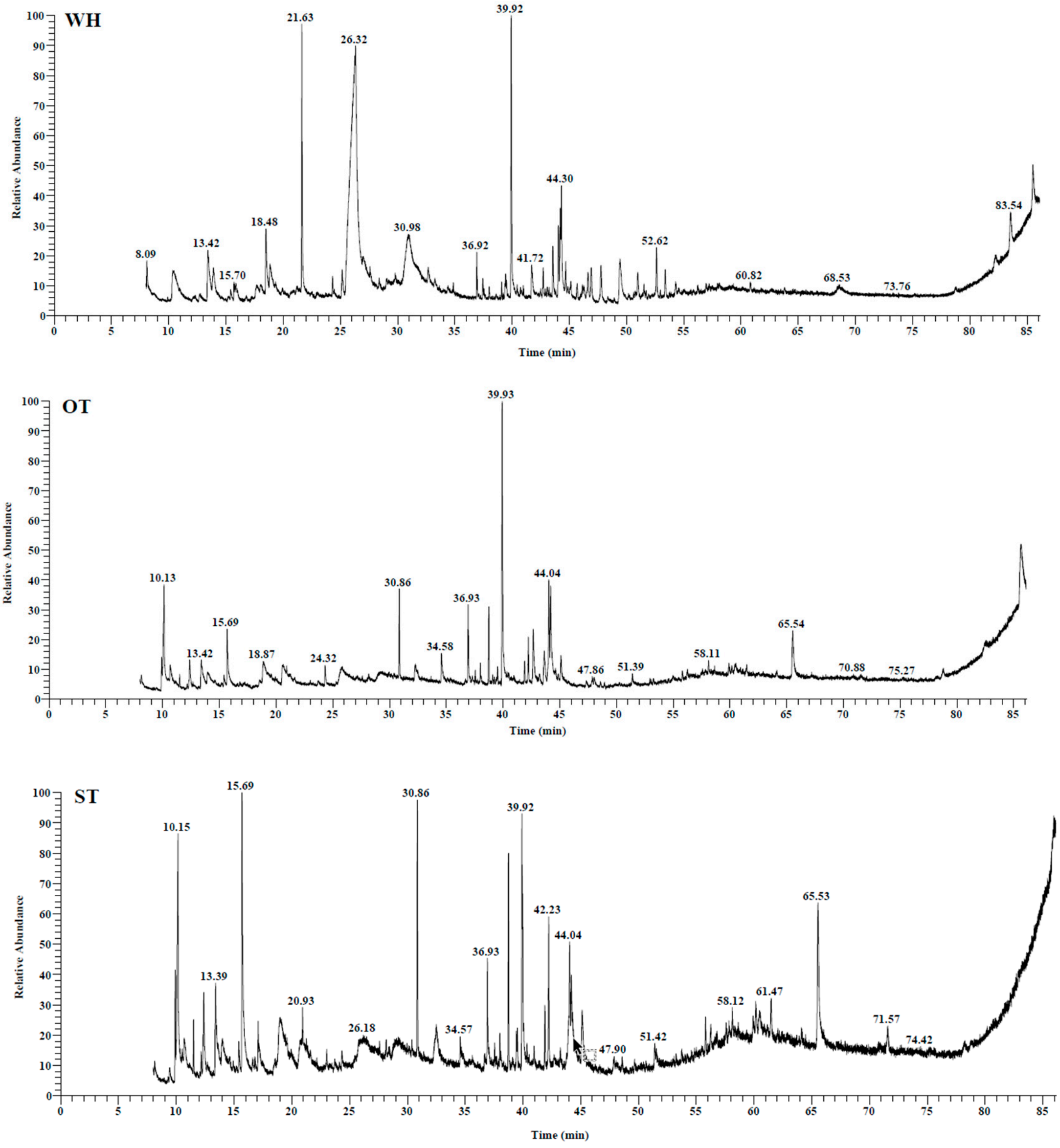

Supplementary Figure S1. GC-MS analysis

Supplementary Figure S2. （A）OPLS-DA analysis of three *H. serrata*. (B) A permutation test of

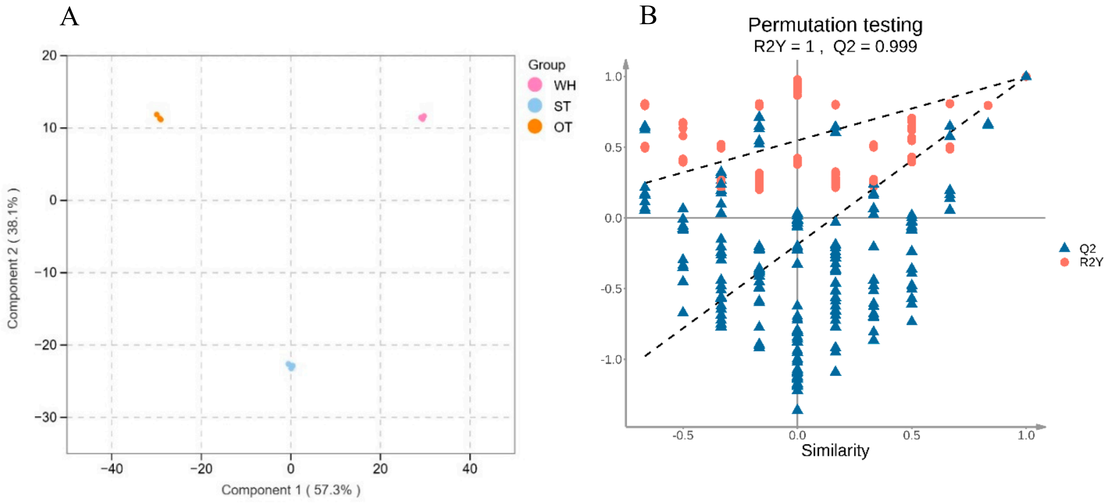

OPLS-DA analysis.

In Fig. 2A, each point represents an individual sample. The proximity between points indicates similarity in metabolite expression patterns, while greater distances reflect larger metabolic differences. Additionally, the confidence intervals is too small to nearly invisible. It suggests high intra-group consistency.
